# Metabolic Interaction of *Helicobacter pylori* Infection and Gut Microbiota

**DOI:** 10.3390/microorganisms4010015

**Published:** 2016-02-16

**Authors:** Yao-Jong Yang, Bor-Shyang Sheu

**Affiliations:** 1 Departments of Pediatrics, National Cheng Kung University Hospital, Medical College, National Cheng Kung University, #138 Sheng Li Road, Tainan 70428, Taiwan; yaojong@mail.ncku.edu.tw; 2 Institute of Clinical Medicine, Medical College, National Cheng Kung University, Tainan 70428, Taiwan; 3 Department of Internal Medicine, National Cheng Kung University Hospital, College of Medicine, National Cheng Kung University, #138 Sheng Li Road, Tainan 70428, Taiwan

**Keywords:** *H. pylori*, microbiota, metabolic interaction, insulin resistant, diabetes

## Abstract

As a barrier, gut commensal microbiota can protect against potential pathogenic microbes in the gastrointestinal tract. Crosstalk between gut microbes and immune cells promotes human intestinal homeostasis. Dysbiosis of gut microbiota has been implicated in the development of many human metabolic disorders like obesity, hepatic steatohepatitis, and insulin resistance in type 2 diabetes (T2D). Certain microbes, such as butyrate-producing bacteria, are lower in T2D patients. The transfer of intestinal microbiota from lean donors increases insulin sensitivity in individuals with metabolic syndrome, but the exact pathogenesis remains unclear. *H. pylori* in the human stomach cause chronic gastritis, peptic ulcers, and gastric cancers. *H. pylori* infection also induces insulin resistance and has been defined as a predisposing factor to T2D development. Gastric and fecal microbiota may have been changed in *H. pylori*-infected persons and mice to promote gastric inflammation and specific diseases. However, the interaction of *H. pylori* and gut microbiota in regulating host metabolism also remains unknown. Further studies aim to identify the *H. pylori*-microbiota-host metabolism axis and to test if *H. pylori* eradication or modification of gut microbiota can improve the control of human metabolic disorders.

## 1. Introduction

The human gut, including the stomach and intestine, is inhabited by a vast number of microorganisms that host microbiota [1]. Gut microbiota starts after birth through contact with the mother’s vaginal, skin, and fecal microorganisms [2,3] and its ecology is influenced by the delivery type, maternal diet, gestational age, and antibiotic exposure [4,5,6]. Using new metagenomics techniques, researchers have demonstrated that gut microbiota regulate the host immune homeostasis and are related to many human metabolic disorders [7]. In an animal study, the gut microbiome increased the capacity of obese mice to harvest energy from their diet [8]. Moreover, the transmission of microbiota from obese mice to germ-free mice led to increased total body fat [8]. Thus, the integrity and balance of gut microbiota play a major role in the metabolic interaction between the host and the microbial community.

*H. pylori* can persistently colonize the gastric epithelium by interacting with bacterial adhesion molecules and gastric receptors [9,10]. The metabolic consequences of *H. pylori* infection have been reported to change the microbial-origin fatty acid and lipid profiles in host blood [11]. In an animal study that used NMR-based metabolic analysis, *H. pylori* infection was shown to disturb the carbohydrate and amino acid metabolism of the host [12]. In addition, metabolic changes are associated with the diversity of gut microbiota. Taken together, these studies indicate that the homeostasis and systemic metabolism between gut microbiota and the host may be altered by *H. pylori*. However, the exact mechanism remains unclear. This review article focused on the metabolic interaction between gut microbiota and *H. pylori*, as well as the related consequences of such interaction on the host’s health.

## 2. Crosstalk between Microbiota and Intestines

Gut microbial communities are known to be inherited from the mother [2,3]. Several studies have shown that human health is susceptible to interactions between gut microbiota diversity or composition and intestinal cells [1,3]. Mucosal and immune homeostasis can be established by a balanced interaction between microbial signals and host immune cells [2,13]. In mice studies, Ivanov *et al*. demonstrated that a single segmented filamentous bacterium can induce effector T cell differentiation in the lamina propria [14]. Other reports show that certain commensal bacteria modulate intestinal inflammatory responses by inducing regulatory T (Treg) cells and the downstream production of transforming growth factor-β (TGF-β) and interleukin-10 (IL-10) [15,16]. Mortha *et al.* reported that intestinal macrophages could sense microbial signals to induce the RORγt^+^ innate lymphoid cells (ILCs) to produce colony-stimulating factor 2 (CSF2), thereby promoting intestinal homeostasis [13]. All of these suggest that autoimmune diseases caused by the innate-adaptive immune activation are likely to be affected by the microbial environment [17].

## 3. Metabolic Roles of Gut Microbiota

Crosstalk and signaling between the host and microbiota occur at both the cellular and metabolic levels. Ktsoyan *et al.* showed that significant concentrations of microbial long chain fatty acids (LCFAs) were present in human blood and corresponded to specific microbial compounds [11]. Furthermore, the profile of these LCFAs is distinguished between healthy and pathologic states. Commensal microbiota can break down indigestible polysaccharides in the diet, thereby serving as 70% of the energy source. Using genomic analysis, studies revealed that human colonic microbes like *Bacteroides* and *Bifidobacterium* possessed abundant polysaccharides and starch breakdown genes [18,19,20].

**Figure 1:**
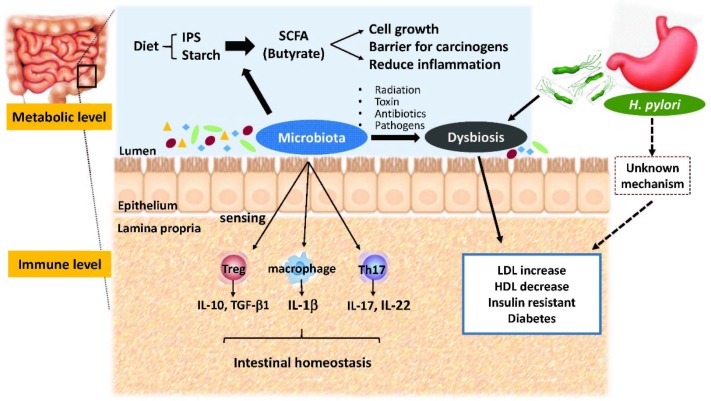
The metabolic and immunologic roles of gut microbiota and *H. pylori* infection on human metabolic disorders. IPS, indigestible polysaccharides; SCFA, short-chain fatty acid; LDL, low-density lipoprotein; HDL, high-density lipoprotein.

The other important energy source of bacterial colonizers of the epithelium, especially butyrate fermenting bacteria, is short chain fatty acids (SCFA) [21]. Microbe-producing butyrate may serve as nutrients for cell growth but also as augmentation of the barrier function to prevent carcinogenesis of the colonic epithelium [22,23]. Using the 16S rRNA sequencing method, Pryde *et al.* demonstrated that the most common butyrate-producing bacteria were *Clostridium* spp., particular in clusters XIVa and IV in the human feces [21]. Decreased production of SCFAs can also be correlated to colonic inflammation and clinical diseases. Kelly *et al.* revealed that microbe-derived SCFAs, particularly butyrate, stimulated epithelial metabolism and decreased intracellular O_2_, resulting in the stabilization of the transcription factor hypoxia-inducible factor-1 (HIF-1) and epithelial barrier function [22]. In a rat model of colon cancer, Mclntyre *et al.* reported that rats fed a high butyrate-producing fiber diet (wheat bran) had significantly fewer tumors and less tumor mass than those given low butyrate-production fiber diet [23]. These results imply that gut microbiota influences local and systemic metabolites, and closely determinates immunity and other protective mechanisms in humans (Figure 1).

## 4. Gut Dysbiosis and Human Metabolic Disorders

An altered balance between gut microbiota and the host contributes to a spectrum of immune, inflammatory, and metabolic disorders. A metagenome-wide association study using deep shotgun sequencing of the gut microbial DNA demonstrated that type 2 diabetes (T2D) in Chinese patients had moderate degrees of gut microbiota dysbiosis, particularly decreased butyrate-producing bacteria [24]. Based on the close association between microbiota and diabetes, Vrieze *et al*. transferred intestinal microbiota from lean donors to recipient males with metabolic syndrome. Six weeks later, the insulin sensitivity of the recipients increased along with levels of butyrate-producing intestinal microbiota [25].

Wen *et al.* used a type 1 diabetes (T1D) non-obese diabetic (NOD) mice model to suggest that signaling through the MyD88 adaptor was critical for T1D development. This effect depended on commensal microbes because germ-free MyD88-negative NOD mice developed robust diabetes [26]. Moreover, the transplantation of microbiota from specific pathogen-free MyD88-negative NOD donors to germ-free NOD recipients attenuated the T1D. Taken together, alterations in intestinal microbiota are associated with insulin resistance and diabetes. The restoration of “healthy microbiota” (microbiota in healthy condition) may be a promising therapeutic strategy for controlling metabolic syndrome.

## 5. *H. pylori* Infection and Metabolic Diseases

Identified by Marshall and Warren in 1984, *H. pylori* can cause chronic gastritis and peptic ulcer disease [27,28]. The World Health Organization (WHO) has categorized *H. pylori* as a group I carcinogen, emphasizing its association with gastric cancer [29]. *H. pylori* have also been associated with several extra-gastric diseases like iron deficiency anemia, idiopathic thrombocytopenic purpura, and childhood growth [30,31,32]. Although the exact relationship between *H. pylori* and diseases is still being debated, bacterial eradication results in long-term benefits [33,34]. Recently, studies demonstrated that *H. pylori* infection was also related to lipid and glucose metabolism [35,36]. A large-scale cross-sectional study revealed that males who were *H. pylori*-seropositive exhibited significantly higher low-density lipoprotein (LDL) cholesterol levels and significantly lower high-density lipoprotein (HDL) cholesterol levels than *H. pylori*-seronegative subjects [37]. Jia *et al.* suggested that lower HDL cholesterol was associated with *H. pylori* infection but did not correlate with the severity of coronary atherosclerosis [38]. A randomized clinical trial is needed to prove that the eradication of *H. pylori* can prevent or improve hyperlipidemia and atherosclerosis.

The influence of *H. pylori* on the host’s glucose metabolism remains controversial. In a hospital-based study, dyspeptic patients with chronic *H. pylori* infection had significantly higher HbA1c level and type 2 diabetes in participants older than 65 years of age, but had decreased insulin secretion and sensitivity in participants younger than 45 years old [39]. A large-scale cross-sectional National Health and Nutrition Examination Survey (NHANES) study revealed that *H. pylori* infection was associated with higher HbA1c, but not self-reported diabetes [40]. However, this correlation was not present in healthy Lebanese adults [41].

The underlying mechanisms of *H. pylori*-induced insulin resistance and diabetes development were studied in a mice model. Zhou *et al*. reported that *H. pylori* infection caused hepatic insulin resistance by mediating the c-Jun/miR-203/SOCS3 signaling pathway [42]. However, there is a paucity of clinical trials that identify the efficacy of *H. pylori* eradication on glycemic control. Vafaeimanesh *et al*. treated 93 consecutive patients with type 2 diabetes mellitus (T2DM) and found that *H. pylori* eradication had no role in glycemic control [43]. In contrast, Bégué *et al*. suggested that the eradication of *H. pylori* in T1DM patients could lead to a short-term decrease in HbA1c level [44]. However, despite these suggestions, testing and treating *H. pylori* in patients with insulin resistance and diabetes are still not routinely done in clinical practice. Further large-scale investigations are needed to clarify the association of *H. pylori* and diabetes.

## 6. *H. pylori* Infection and Gut Microbiota

*H. pylori* and gut microbiota both influence the host’s metabolism. Stearns *et al.* studied bacterial bio-geography from different GI sites in four healthy volunteers. Their results revealed larger Shannon and phylogenetic diversities in mouth samples than in samples from the stomach, duodenum, and colon [45]. They also identified that stomach microbes were the least diverse compared than those from the colon and stool. The impact of *H. pylori* infection on changes in stomach or colon microbiota has been studied in animals and humans [46,47,48,49,50]. Martin *et al*. investigated stomach microbes by deep sequencing of 16S-rRNA in rhesus macaque pre- and post-inoculation of *H. pylori*. They found no significant difference in the average relative abundance of non-*Helicobacter* taxa in the antrum or corpus [46]. A culture method applied by Yin *et al.* demonstrated an increment in *Enterococcus* spp. and *Staphylococcus aureus* in the stomach and duodenum, and a downshift of *Lactobacillus* spp. in the stomach after *H. pylori* inoculation in Mongolian gerbils [47]. In the same model, Heimesaat *et al.* demonstrated that mice infected with immuno-pathologic *H. pylori* B8 could increase the luminal load of *E. coli* and enterococci in the cecum and of *Bacteroides*/*Prevotella* spp. in the colon [48].

Using deeper sequencing methods, human studies have tried to determine the differences in gastric microbiota between *H. pylori*-infected and non-infected persons [49,50]. Bik *et al.* found that the gastric bacterial composition is similar between *H. pylori-*infected and non-infected subjects at the phylotype level [49]. In contrast, Maldonado-Contreras *et al.* reveal marked differences in the structure of gastric bacterial community in *H. pylori*-infected stomach, with an increase in *Proteobacteria* and a decrease in *Actinobacteria*, *Bacteroidetes*, and *Firmicutes* [50]. Since the association between *H. pylori* infection and changes in gastric and colonic microbiota is still being debated, more studies on the influence of interactions between commensal colonization and the microbiota–host–environment are critical for understanding how *H. pylori* influences the gut microbiota and human health [51,52].

## 7. Inflammatory Bowel Diseases and Intestinal Microbiota

Crohn’s disease (CD) and ulcerative colitis (UC) are both immune-related chronic inflammatory bowel diseases (IBD). Clinical trials have shown that a dysbiosis of fecal microbiota, including changes in microbial diversity and an abundance of certain genus are closely related to the development and maintenance of IBD in adults and children [53,54,55,56]. The diversity of microbiota includes a significant decrease in the genus *Faecalibacterium* and an increase in the genus *Bacteroides* in adults with CD and active disease [53]. Unlike findings in adults, there is less abundance and diversity of the bifidobacterial population in children with IBD [55], implying a close relationship between intestinal microbiota and human IBD. Thus, re-establishing gut homeostasis in patients with IBD is desired in order to improve the disease severity and prevent relapse.

Recurrent and serious *Clostridium difficile* infections have good responses in colonic and frozen-capsulized fecal microbiota transplantation (FMT) [57,58]. Moayyedi *et al*. reported that FMT enema once weekly for six weeks significantly improved disease remission by week 7 in patients with UC (24% *vs*. 5%) [59]. Moreover, fecal donor and disease duration can affect outcomes. Although this trial is an original study, some limits, such as the duration, route, and dosage of FMT administration, as well as donor selection, still warrant further clarification.

## 8. Inflammatory Bowel Diseases and *Helicobacter* Infection

If *H. pylori* infection and IBD are both related to changes in gut microbiota, is there a link between *H. pylori* infection and IBD through alterations in gut microbiota? Luther *et al.* conducted a meta-analysis and concluded a significantly lower rate of *H. pylori* infection in IBD patients than in controls (27.1% *vs.* 40.9%), with a relative risk of 0.64 (95% confidence interval [CI]: 0.54–0.75) [60]. The potential mechanisms of *H. pylori* protection in IBD were fully discussed by Papamichael *et al*. [61]. Like genetic and environmental factors, *H. pylori* can induce a Treg response and down-regulate the pro-inflammatory Th1/Th17 pathway on the gastric mucosa and in peripheral blood [61,62,63].

Theoretically, such immune changes may be beneficial in protecting against the development of CD, a characteristic disease that up-regulates type 1 helper-T immune responses [64]. In animal studies, administration of *H. pylori* DNA or live bacteria to experimental mice can ameliorate colonic inflammation induced by dextran sulfate sodium (DSS) and *Salmonella*, respectively [65,66]. Protection against DSS-induced colitis in mice is dependent on NLRP3 inflammasome and interleukin-18 signaling [67]. However, to answer the causative and protective roles of *H. pylori* infection against IBD, evidence on the increased development of IBD in patients after the eradication of *H. pylori* is needed.

Recent studies focused on the association between non-*H. pylori* *Helicobacter species* (NHPH) and human gastrointestinal diseases, including Crohn’s disease, hepatobiliary disease, and gastritis [68,69,70]. Entero-hepatic *Helicobacteraceae* were detected by PCR sequencing in 59% of fecal samples from children with Crohn’s disease, compared to 9% in healthy controls [69]. Nonetheless, among 160 Chinese patients with inflammatory bowel disease (10%) and 80 controls (6.3%), there was no difference in the detection rate of *Helicobacter* spp. in intestinal specimens [70]. The role of gut microbiota in humans, with focus on the interaction between *H. pylori* and IBD, should be investigated in the future. Nagalingam et al. reported that *H. hepaticus* triggered colonic inflammation only in conventional raised IL-10^−/−^ C57BL/6 mice, but not in germ-free mice [71], implying that conventional microbiota are essential for *H. hepaticus*-induced colonic inflammation. Moreover, *H. hepaticus* infection altered the structure of intestinal microbiota even with prior antibiotic treatment.

Interestingly, Yang *et al.* demonstrated that *H. hepaticus*-induced colonic inflammation was different in the IL-10^−/−^ C57BL/6 mice obtained from two facilities. They further revealed different susceptibilities of *H. hepaticus*-induced colonic inflammation that originated from distinct microbiota among the groups. Further studies are therefore warranted in order to compare the discrepancy in microbiota structure between pre- and post-*H. pylori* eradication, and to investigate the structure susceptible to IBD development after *H. pylori* eradication.

## 9. Metabolic Interaction of *H. pylori* and Gut Microbiota

Although numerous studies suggest that gastric and colonic microbiota communities are changed after *H. pylori* infection [12,47,50], only one animal study has shown that *H. pylori* alters gut microbiota, with simultaneous changes in carbohydrate and amino acid metabolisms [12]. Recently, Khosravi *et al.* demonstrated that *H. pylori*-infected specific pathogen-free (SPF) mice had elevated levels of leptin, insulin, and peptide YY metabolites. Nonetheless, their growth curves remained inconclusive. Interestingly, germ-free mice with *H. pylori* infection also have increased levels of ghrelin and peptide YY, but with concomitant weight loss and malnutrition [72]. Experience has shown that *H. pylori* infection alters the composition of fecal *Bifidobacterium*/*E. coli* in children, which, in turn, is improved by ingesting probiotics-containing yogurt [73]. Lastly, the eradication of *H. pylori* increases the growth in weight and height among children, together with increased serum acylated ghrelin level [32]. These findings suggest that *H. pylori* infection alters host metabolism and that commensal microbiota can attenuate the growth delay caused by *H. pylori*. The detailed mechanisms of metabolic interaction between *H. pylori* and gut microbiota in hosts warrant more studies.

## 10. Conclusions

Both *H. pylori* and gut microbiota regulate the host metabolism. Some clinical observations and animal studies support the relationship of *H. pylori* infection and dysbiosis of gut microbiota to metabolic disorders like insulin resistance and diabetes [24,25,26]. However, how *H. pylori* and gut microbiota interact with each other to regulate the host metabolism remains unknown. Further studies are warranted to identify the *H. pylori*–microbiota–host metabolism axis and to test if the eradication of *H. pylori* or the modification of gut microbiota can control or treat human metabolic disorders.
